# Chemical Modulation of Optical Forces for Dynamic Light-Driven Materials

**DOI:** 10.1021/acsphotonics.6c00535

**Published:** 2026-07-31

**Authors:** Laia Torrella-Adriaensen, Boris Louis, Jagannath Satpathy, Anna Tarrats-Duran, Jose Rodrigo Magaña, Cristina Fornaguera, Santi Nonell, Susana Rocha, Johan Hofkens, Roger Bresolí-Obach

**Affiliations:** † Institut Químic de Sarrià, 16522 Universitat Ramon Llull, Via Augusta 390, Barcelona 08017, Spain; ‡ Department of Chemistry, 54517 Katholieke Universiteit Leuven, Celestijnenlaan 200F, Heverlee 3001, Belgium

**Keywords:** optical forces, pH-responsive materials, functional materials, optical matter, absorption force

## Abstract

Controlling colloidal assembly with high spatial and temporal precision is essential for next-generation photonic and functional materials. Optical forces offer unique advantages, yet remain largely decoupled from chemical stimuli. Here, we introduce a simple strategy to chemically gate absorption-driven optical forces using pH-responsive polyaniline-doped polystyrene microparticles. Under basic conditions, enhanced absorption at 561 nm amplifies axial optical forces, producing superdiffusive motion and promoting the formation of large, ordered interfacial assemblies. Instead, acidic conditions suppress absorption, yielding only small, disordered clusters. This reversible chemical control establishes a general framework for coupling light-matter interactions with chromogenic chemistry, enabling chemo-adaptive, light-driven materials for dynamic sensing and programmable matter.

## Graphical abstract

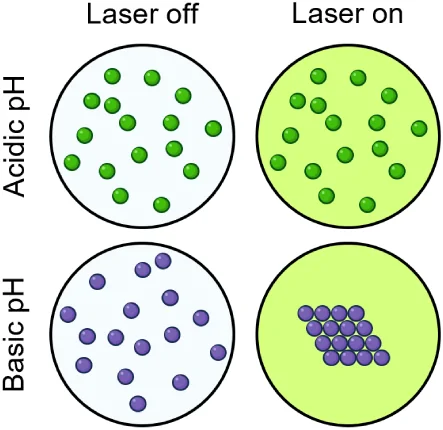

## 1 Introduction

The self-organization of colloidal nano- and microscale objects into intricate or periodic structures underlies the development of next-generation photonic and functional materials (e.g., photonic crystals, superlattices). Mastering the particle–particle interactions that drive these structured assemblies is therefore a central challenge, opening the way to rationally engineered materials with unique optical, electrical, and mechanical properties.^1–5^ Several physicochemical strategies have been developed to modulate these interactions, including controlling particle shape or surface functionality, generating chemical gradients or hydrodynamic effects, aligning liquid crystals, or applying external electric or magnetic stimuli.^6–9^ Despite these advances, these methods lack the spatiotemporal precision necessary for effective and selective control at the microscale, particularly when assembling large numbers of particles.^10–12^

To address these limitations, optical forces (e.g., scattering, gradient, and absorption forces) offer an exceptional route to control matter with unmatched spatial and temporal precision, acting exclusively where and when light is applied.^13–17^ When combined with complementary nonoptical interactions – such as electrostatic interactions, collective hydrodynamic interactions, capillary forces, thermophoretic effects, or Marangoni forces – light becomes a powerful sculpting tool capable of directing colloidal behavior far beyond simple optical trapping.^18–21^ Such synergistic interactions have given rise to the formation of optical matter, a type of active colloidal self-assembly, where light-driven forces organize colloidal particles into ordered, reconfigurable assemblies that extend far beyond the directly illuminated region.^22–26^ The ability to shape, stabilize, and reversibly reconfigure these assemblies in real time, through controllable light parameters (e.g., beam shape, size, polarization), establishes optical matter as a powerful platform for creating adaptive and programmable materials.^27–30^

One optical force of particular interest is the absorption force, an essential yet often-overlooked component of optical forces.^31–33^ This force occurs when an electronic transition within the particle (typically S_0_ → S_n_) resonates with incoming photon flux, such that the absorbed photons transfer their momentum to the particle.^16,34,35^ For particles in solution, the impact of the absorption force is limited, as it only amplifies the total exerted force by approximately 10–35%. At an interface, however, the effect becomes remarkable, with trapping stiffness enhanced by over four times due to a combined effect of the optical resonance effect and the barrier imposed by the interface.^36^ Control over the absorption force has been further optimized by leveraging nonlinear optical effects, such as forces from excited-state transitions and recoil forces due to stimulated emission.^37^ Interestingly, the absorption force is not confined to materials with intrinsic electronic transitions. Non-resonant particles can become resonant when externally bound to dyes, which enables significant force amplification as multiple chromophores resonate simultaneously. The relevance of the absorption force has surged recently, being applied in optical force modulation, particle sorting, manipulation of transport speed, single-particle spectroscopic measurements, and spin rotational control.^16,38–40^ Crucially, the absorption force can be extended beyond single-particle effects and, at interfaces, can modulate interparticle interactions through coupling with nonoptical forces, thereby leading to the formation of ordered multibody assemblies.^41,42^

Despite these advances, no existing studies on optical forces have demonstrated the ability to modify – let alone control – particle–particle interactions in a way that predictably alters the morphology of these self-assembled structures.^43^ Achieving this level of control calls for an approach in which optical forces, while retaining their intrinsic spatiotemporal precision, are gated by a chemical stimulus that can be addressed independently of the optical field.^1–5,10,44^ Photochromic approaches, such as that reported in Ito et al.,^35^ have shown that changes in molecular absorption can be used to modulate optical forces at the single-particle level. Building on this concept, we previously demonstrated chemical control over optical trapping forces at an interface, using oxygen as the chemical input and achieving optical force modulation through the transient population of a microsecond-lived excited state.^45^ That approach, however, relied on a complex optical implementation, involving nonlinear optical processes and the concurrent use of widefield and tightly focused laser excitation. As a result, it was effective only under conditions of very high photon flux, restricting its applicability to the focal region of a tightly focused beam and limiting its scalability to multibody optical matter. Thus, a practical platform to translate chemical input into control over collective assembly remains lacking.

In this work, we address this gap by designing pH-responsive polyaniline-doped microparticles that act as a chemical gate for absorption-driven optical forces. Polyaniline is a colorimetric, pH-sensitive dye that exhibits a strong electronic transition centered around 575 nm under basic conditions, which is significantly reduced in acidic environments. This reactivity provides a straightforward handle to tune particle absorption and thus the absorption force at both the single-particle and multibody levels. In particular, we connect chemical state to force readout and collective organization, establishing a quantitative pathway from chemically controlled absorption to particle propulsion and assembly behavior. More broadly, extending this concept to other colorimetric chromophores responsive to a wide range of chemical stimuli^46^ should enable a new class of (opto)­chemically adaptive materials whose light-matter interactions dynamically respond to the local chemical environment, thereby opening new avenues in the design of sensors and optically responsive matter.

## 2 Results and Discussion

### 2.1 Synthesis and Characterization of Chromogenic Microparticles

Polyaniline-doped microparticles with pH-responsive optical properties were prepared in a two-step process. First, bare polystyrene microparticles (PS MPs) were synthesized via state-of-the-art free radical dispersion polymerization in ethanol using polyvinylpyrrolidone as a stabilizer.^47,48^ Subsequently, polyaniline-doped PS MPs (PDPS MPs) were obtained by swelling the PS MPs in an acidic aqueous suspension containing aniline, followed by oxidative polymerization using ammonium persulfate to form a polyaniline layer on the particle surface (Figure 1A). The resulting PDPS MPs presented a diameter of approximately 2 μm (Figure 1B) and displayed a monodisperse particle size distribution (Figure 1C). The surface morphology of PDPS MPs exhibited slight roughness attributed to the surface-grafted polyaniline layer (Figure 1B). In addition, as shown in Figure 1D, the PDPS MPs remained stable in different acidic and basic environments, with minimal hydrodynamic diameter variation. These findings confirm the homogeneity and robustness of the PDPS MPs under varying pH conditions, supporting their suitability for further pH-dependent optical force modulation experiments.

**Figure 1:**
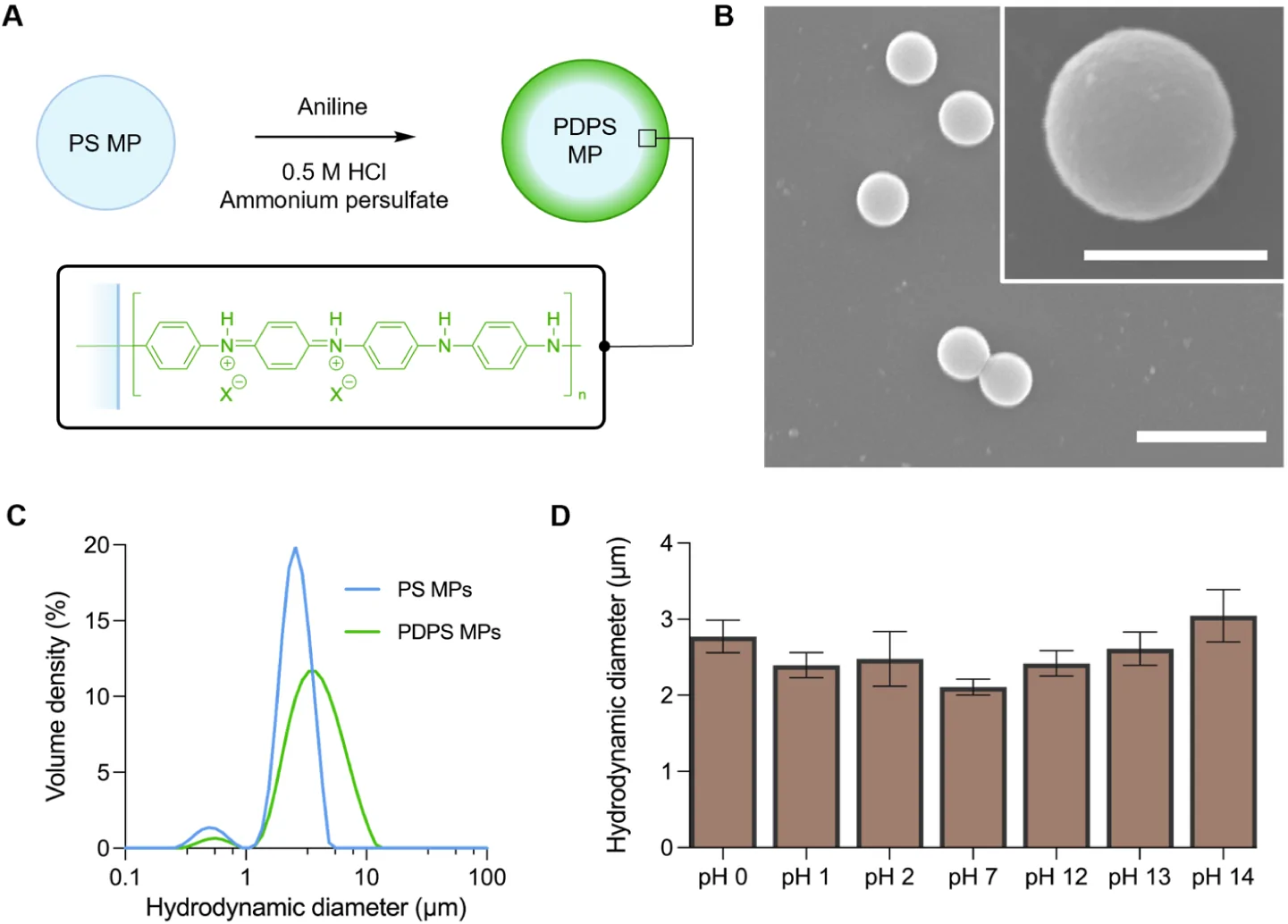
Synthesis and characterization of PDPS MPs. (A) Schematic illustration of the synthesis protocol for PDPS MPs. The inset shows the chemical structure of the polyaniline polymer (emeraldine salt), with counterion X^–^ = Cl^–^. (B) Scanning Electron Microscopy (SEM) image of PDPS MPs (scale bar = 5 μm). The inset shows a magnified view of an individual PDPS MP, highlighting surface roughness (scale bar = 2 μm). (C) Hydrodynamic diameter distribution of PS MPs and PDPS MPs at pH 7. (D) Evolution of the mean hydrodynamic diameter of PDPS MPs under different pH conditions. The error bars represent standard deviation (*n* = 3).

### 2.2 pH-Dependent Optical Absorption

With the PDPS MPs in hand, we studied their light absorption behavior under two extreme pH conditions: pH 2 (acidic) and pH 10 (basic), where polyaniline is found in its emeraldine salt and emeraldine base forms, respectively (Figure 2A). To closely replicate the conditions used in the experiments below, we measured the absorption spectra of the aqueous particle suspensions using a spectrophotometer equipped with an integrating sphere, which effectively removes the contribution from light scattering. As reported in the literature,^49^ a broad absorption band centered around 560 nm appears under basic conditions, corresponding to the emeraldine base form of polyaniline. Upon acidification, protonation converts the emeraldine base into its salt form, extending the conjugation of the system and shifting the absorption bands to longer wavelengths (∼700 nm; Figure 2B). Given the broad absorption profile of the PDPS MPs, which includes multiple electronic transitions, we identified the 500–600 nm range as a region of strong absorption contrast between basic and acidic pH conditions. Based on this, we selected a 561 nm laser line to exert optical absorption forces in all subsequent experiments. Notably, the PDPS MP suspension exhibited no detectable fluorescence at either pH, suggesting a short excited-state lifetime. This observation is consistent with literature reports from ultrafast transient absorption spectroscopy,^50,51^ which describe excited-state lifetimes below 100 ps for both emeraldine salt and base forms. Such short lifetimes enable a higher turnover (i.e., frequency) of resonance cycles per time unit, thereby enhancing the magnitude of the optical resonance force, making the PDPS MPs ideally suited for the formation of assemblies driven by absorption forces.

**Figure 2:**
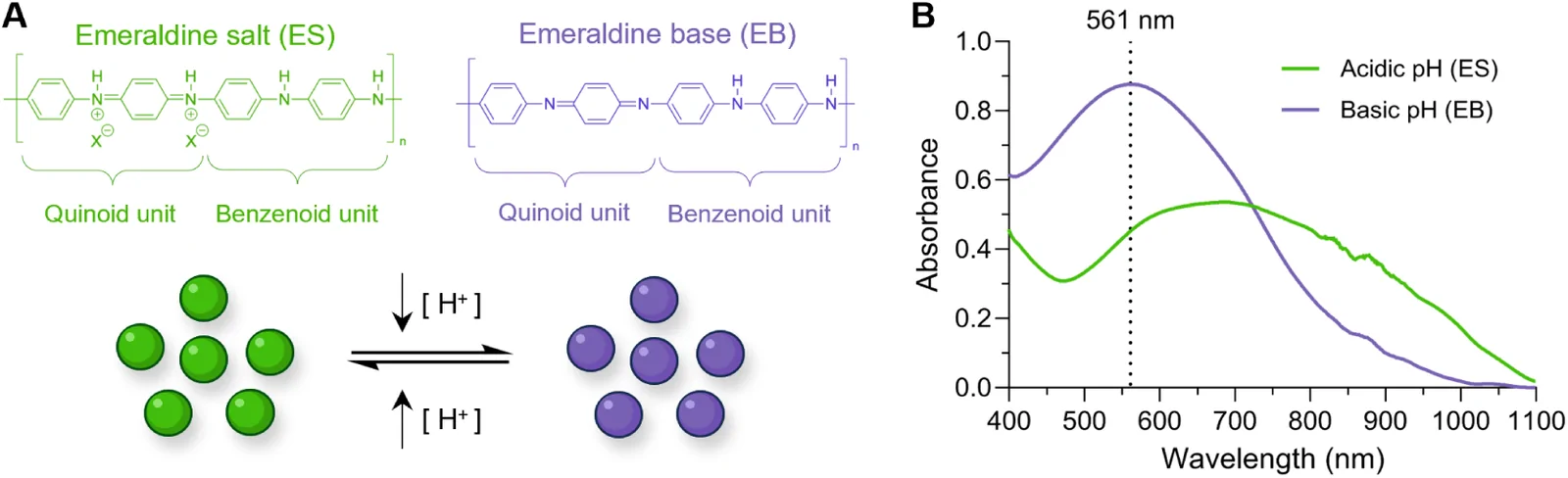
pH-dependent structure and properties of PDPS MPs. (A) Chemical structures of polyaniline in its protonated emeraldine salt (ES) form (green) and its deprotonated emeraldine base (EB) form (purple), showing the reversible transition influenced by pH. (B) Vis-NIR absorption spectra of 2% w/v PDPS MPs suspensions at acidic pH (pH 2, emeraldine salt, green) and basic pH (pH 10, emeraldine base, purple).

### 2.3 Modulation of Absorption Forces at the Single-Particle Level

To investigate how pH modulates the absorption force acting on individual PDPS MP, we tracked their motion in suspension under widefield excitation from a vertically propagating 561 nm laser. At this scale, particle inertia is negligible, and the overall optical force is balanced by viscous drag as described by Stokes’ drag law.^52^ As a result, the directional MP propulsion, calculated as the instantaneous (frame-to-frame) speed, is a direct indicator of the magnitude and direction of the exerted optical force (Figure 3A).

Using an optical multiplane microscope (Supporting Information, Figure S1) in combination with 3D single-particle tracking (SPT) (Supporting Information, Figure S2), we observed distinct directional motion of the PDPS MPs. While average lateral (x- and *y*-directions) speeds remained close to zero, indicative of random Brownian motion, the average axial (*z*-direction) speed was consistently positive across all tested pH conditions (Figure 3B). This unidirectional propulsion along the *z*-axis aligns with the axial propagation of the 561 nm widefield excitation, indicating that the absorption force primarily acts vertically, effectively lifting the PDPS MPs. Control experiments using bare (non-doped) PS MPs showed purely Brownian motion in all three spatial directions, regardless of pH, thereby validating that the observed axial propulsion (i.e., force) in PDPS MPs arises exclusively from polyaniline-mediated optical absorption (Supporting Information, Figure S3). Furthermore, photothermal flows can be excluded, as the maximum temperature increase under the highest irradiance conditions is estimated to be negligible (Δ*T* < 0.3 K; see Supporting Information for details).

Of key importance, axial propulsion speed was significantly higher under basic conditions compared to acidic ones (1.35 versus 0.85 μm/s) (Figure 3B). This 1.6-fold increase in speed is in reasonable agreement with the corresponding increase in absorption at 561 nm (1.9-fold). Thus, this behavior is consistent with the enhanced absorption of PDPS MPs at higher pH, which promotes stronger photon momentum transfer and, consequently, a larger absorption force. Notably, the acidic form of emeraldine salt still absorbs at 561 nm (Figure 2B), which explains the non-zero axial velocity observed under acidic conditions.

Furthermore, as shown in Figure 3B, the axial speed follows a sigmoidal trend as a function of pH, reflecting the pH-dependent optical response of the PDPS MPs. The estimated p*K*_a_ of 3.0 agrees well with reported p*K*_a_ values for the emeraldine salt/base transition, further supporting the mechanistic interpretation of the observed absorption forces.^53^

Further details are obtained by mean squared displacement (MSD) analysis. The MSD of colloidal particles can generally be described by a power-law dependence on the lag time, where the exponent (α) reflects the nature of the motion: purely diffusive Brownian motion yields α = 1, ballistic motion results in α = 2, and confined motion leads to values below 1.^54,55^ As shown in Figure 3C, the MSD of PDPS MPs at pH 10 along the *z*-axis exhibits superdiffusive behavior (α > 1), indicating directed motion and confirming propulsion along the *z*-direction. In contrast, the MSD of the same particles displays an exponent close to unity in the xy-plane, characteristic of Brownian diffusion. This reinforces the role of absorption force: at pH 10, where polyaniline strongly absorbs at 561 nm, irradiation along the *z*-axis propels the particles upward, while motion in the lateral plane remains mainly diffusive. Figure 3D further illustrates the pH-dependent effect of absorption on MSD, as motion along the *z*-axis increases more strongly with lag time at pH 10 than at pH 2. Quantitative analysis yields α = 1.7 at pH 10, while at pH 2, this effect is weaker (α = 1.3). A log–log plot of MSD versus lag time, from which α is directly obtained as the slope, is provided in the Supporting Information (Figure S4). Altogether, these results unequivocally demonstrate that absorption forces (and, therefore, optical propulsion) operating in the linear optical regime can be predictably regulated by a chemical stimulus, here encoded through pH-dependent protonation and the resulting modulation of particle resonance. Indeed, the chemically gated control of optical forces unveils a powerful strategy for embedding chemical responsiveness into optical manipulation, thereby enabling the next generation of reconfigurable, chemically encoded optical matter.

**Figure 3:**
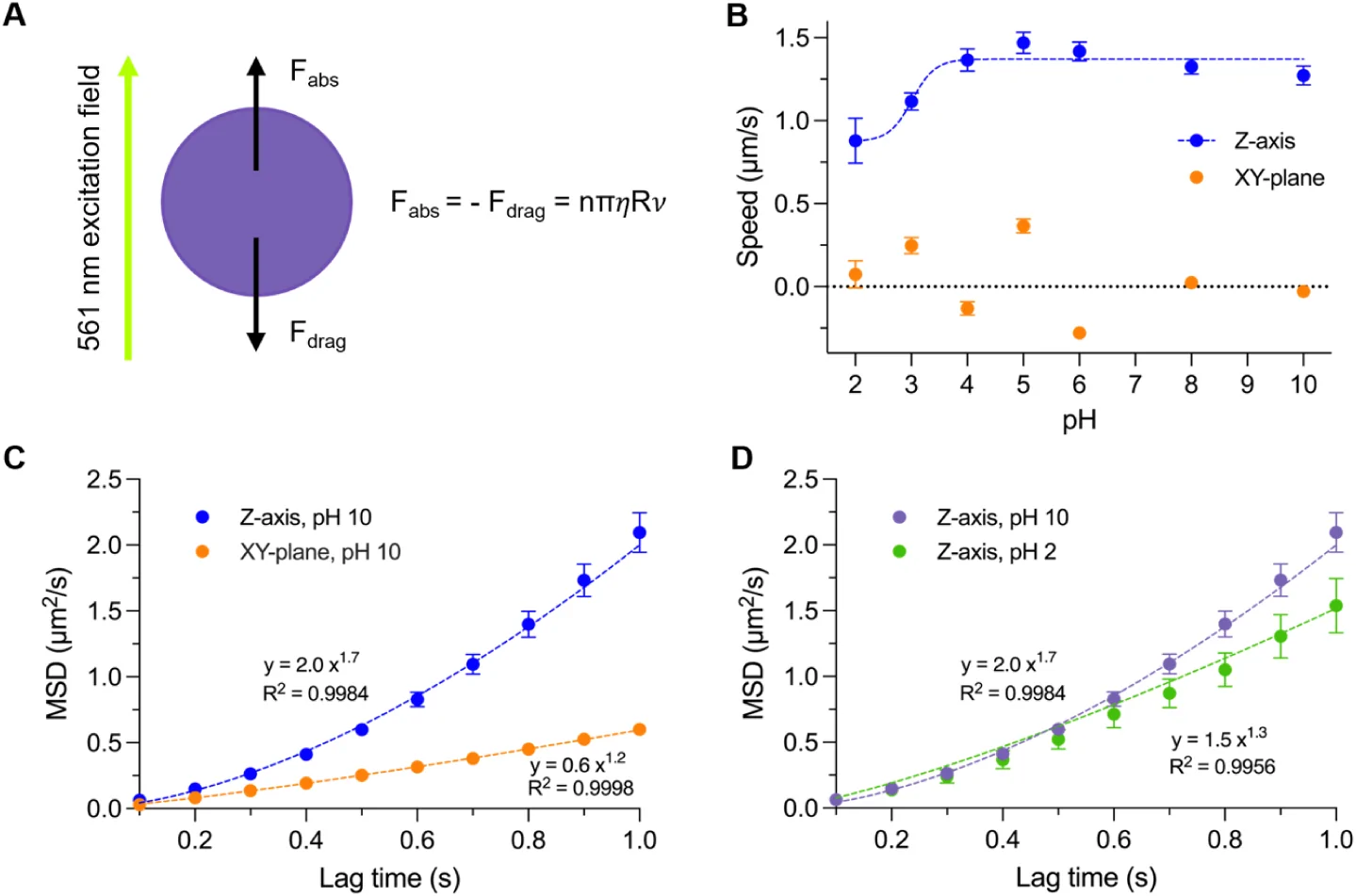
pH-dependent modulation of absorption forces at the single-particle level. (A) Schematic representation of absorption forces acting on a PDPS MP under a 561 nm excitation field. The absorption force is balanced by the viscous drag force, which is described by Stokes’ law, where *n* = 6 for a rigid spherical particle, *η* is the dynamic viscosity of the medium, R is the particle’s radius, and ν is the particle’s speed. (B) Speed of PDPS MPs along the Z-direction (blue) and XY-plane (orange) as a function of pH. The error bars represent the standard error of the mean (SEM). The dashed line corresponds to a sigmoidal fit, with p*K*_a_ = 3.0. (C) Mean squared displacement (MSD) of PDPS MPs at pH 10 along the *Z*-direction (blue) and within the XY-plane (orange) as a function of time. The error bars represent the SEM. The dashed lines correspond to power-law fits, with the equation and goodness of fit (R^2^) indicated in the figure. (D) Mean squared displacement (MSD) of PDPS MPs along the *Z*-direction measured at pH 10 (purple) and pH 2 (green) as a function of time. The error bars represent the SEM. The dashed lines correspond to power-law fits, with the equation and goodness of fit (R^2^) indicated in the figure.

### 2.4 Chemical Control of Optical Matter

With the knowledge that the PDPS MPs exhibit pH-dependent optical absorption, and based on our previous work on absorption force-driven assemblies,^41,42^ we next investigated the formation of multibody optical matter systems. We have shown that the absorption force, in conjunction with capillary and hydrodynamic interactions, can induce the formation of optical matter in the form of hexagonal close-packed (HCP) assemblies at the D_2_O/air interface^41^ (Figure 4A). In D_2_O, the particles are naturally positioned near the interface due to buoyant forces. When subjected to upward-directed absorption force, the particles deform the interface at multiple points. These deformations generate attractive capillary forces that promote the self-assembly of particles into ordered structures (Figure 4B).

Accordingly, concentrated suspensions of PDPS MPs exposed to 561 nm excitation under basic pH conditions (where the absorption force is strongest) formed large, well-defined HCP ordered optical matter assemblies (Supporting Information, Movie S1 and Movie S2). In contrast, under identical irradiation conditions at acidic pH, only small, disordered aggregates were observed (Supporting Information, Movie S3 and Movie S4). To quantify these differences in behavior, we analyzed the size and distribution of assemblies, defined as clusters comprising more than two particles. Under acidic pH, assembly growth was strongly limited, with a maximum cluster size of 5 MPs. In contrast, under basic conditions, the assemblies displayed a broader size distribution, with some clusters containing more than 20 MPs (Figure 4C). This distinction was also reflected in the overall particle distribution: at acidic pH, 55% of MPs remained isolated and did not participate in any assembly, 25% formed dimers, and only 20% formed larger clusters (of 3, 4, or 5 MPs). Conversely, under basic pH conditions, 74% of MPs were found in assemblies (58% of the MPs in assemblies larger than five MPs), with only 17% remaining as single MPs (Figure 4D). Control experiments using bare PS MPs failed to produce assemblies under all tested conditions,^41^ confirming that the absorption force is the key element behind the formation of these HCP assemblies.

**Figure 4:**
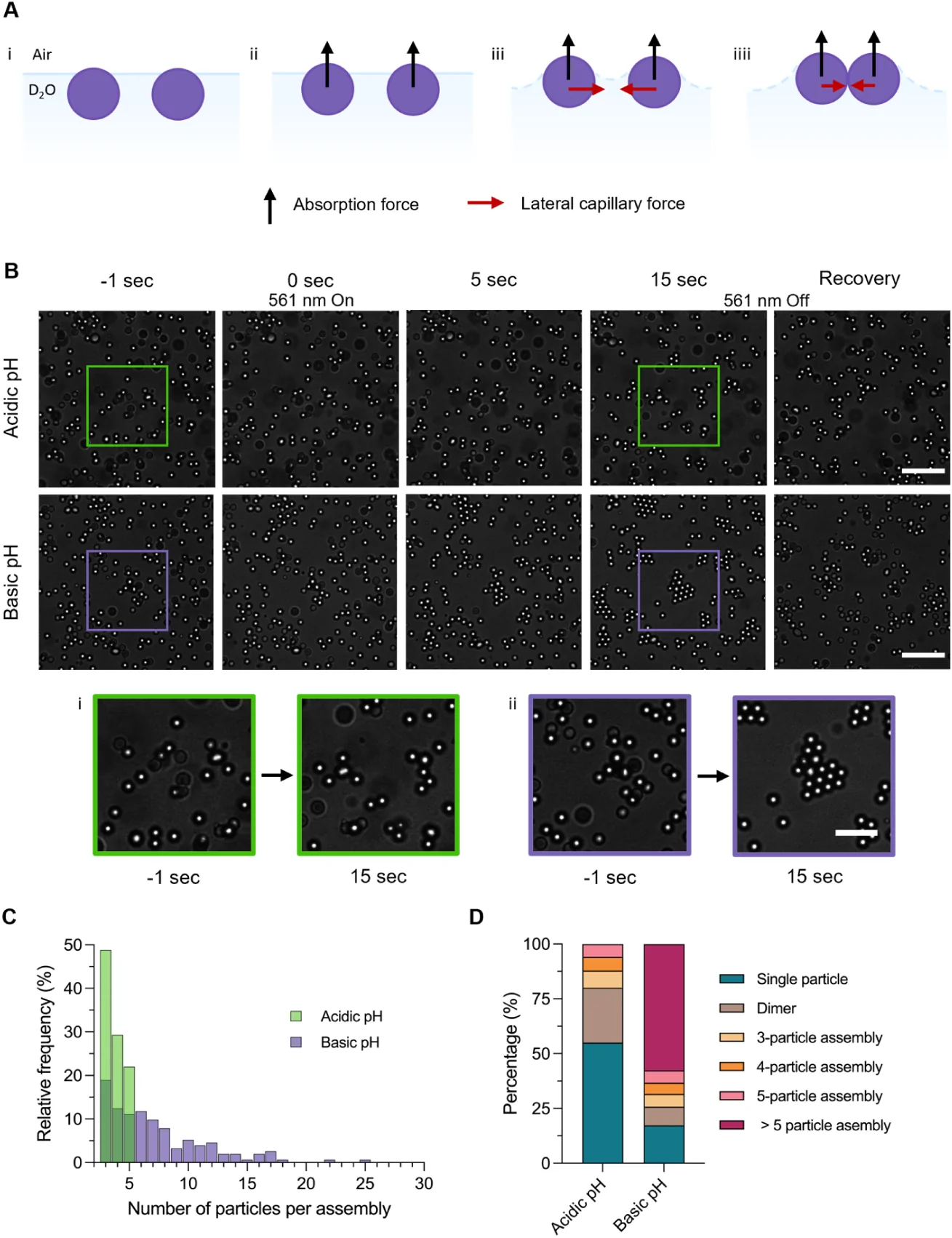
pH-dependent formation of optical matter from PDPS MPs. (A) Schematic representation of PDPS MP assembly at the air/D_2_O interface driven by absorption force. Black and red arrows indicate absorption forces and capillary interactions, respectively. (B) Time-lapse brightfield microscopy images showing the dynamic assembly behavior of PDPS MPs under acidic (pH 2, top row) and basic (pH 10, bottom row) conditions. Images are captured before switching on the laser (-1 sec), during 561 nm laser irradiation at 114 W/cm^2^ (0, 5, 15 sec), and after switching off the laser (Recovery) (scale bar = 20 μm). The insets show magnified views at (i) acidic pH (pH 2) and (ii) basic pH (pH 10) before (-1 sec) and after 15 s (15 sec) of laser irradiation (scale bar = 10 μm). (C) Histogram depicting the relative frequency of different assembly sizes under acidic (green) and basic (purple) pH conditions. (D) Percentage distribution of particles across different assembly sizes under acidic and basic pH conditions (*n* > 750).

To further study this relationship, we next examined the response of PDPS MPs as a function of irradiance. At the single-particle level, increasing the irradiance produced a linear increase in axial speed (Supporting Information, Figure S5), as higher irradiance increases the number of photons absorbed, thereby enhancing momentum transfer and the resulting absorption force. This power-dependent response was also evident at the multibody level. Under basic conditions, increasing the irradiance accelerated the onset of assembly and produced larger clusters after a given irradiation time (Supporting Information, Figure S6). This behavior is consistent with a stronger absorption force, which induces earlier and larger deformation of the D_2_O/air interface, thereby enhancing capillary attraction and promoting faster recruitment of surrounding MPs into the assemblies. In contrast, under acidic conditions, where the absorption at 561 nm is weaker but non-zero, assembly formation required substantially higher power. Whereas an irradiance of 56 W/cm^2^ was sufficient to induce assembly within 15 s under basic conditions, under acidic pH small assemblies only began to emerge at 133 W/cm^2^ and became more pronounced at longer irradiation times or higher laser irradiances (Supporting Information, Figure S7). Thus, achieving comparable assembly at acidic pH requires approximately twice the irradiance used for basic conditions, in line with the lower optical absorption (absorption ratio of 1.9 between basic and acidic pH at 561 nm). These observations indicate that the pH-dependent differences in assembly behavior are primarily driven by differences in absorption-force magnitude rather than by a purely electrostatic origin.

We next investigated whether the collective assembly response follows the intermediate protonation state expected near the emeraldine salt/base transition. At pH 3, which corresponds to the p*K*_a_ determined from the single-particle measurements, PDPS MPs displayed a clearly intermediate behavior (Supporting Information, Figure S8): assemblies still formed, but they appeared later and remained smaller than those observed under conditions above the p*K*_a_, such as pH 5 and pH 10. This intermediate response links the multibody assembly behavior to the gradual pH-dependent modulation observed at the single-particle level and shows that assembly is not a simple binary switch but instead correlates with the protonation state of the polyaniline across the transition.

Finally, we assessed the reversibility of the assembly process along its two independent control axes, optical and chemical. Optical reversibility was shown through repeated laser on/off cycling: the PDPS MPs assembled under irradiation and disassembled once the laser was switched off. This response was reproduced over at least six consecutive cycles without noticeable loss of assembly efficiency (Supporting Information, Figure S9 and Movie S5). Chemical reversibility was demonstrated by sequentially switching the sample between pH 2 and pH 10 during sample preparation, followed by extraction of aliquots for imaging after each condition. Across six pH-switching steps, comprising three acidic and three basic states, the assembly behavior remained reversible: every basic state yielded assemblies upon irradiation, whereas every acidic state suppressed assembly formation under identical irradiation conditions (Supporting Information, Figure S10 and Movie S6). We observed no detectable hysteresis, and neither the rate nor the extent of assembly degraded over the cycles examined. These results show that the system can be reconfigured repeatedly by both optical and chemical means, a reconfigurability that is central to its potential as a dynamic platform.

All in all, these results demonstrate that chemical modulation of optical absorption can effectively control the formation of optical matter at the multibody level, unlocking a powerful strategy for designing chemo-adaptive light-driven self-assembly systems.

### 2.5 Discussion: Chemical Gating as an Orthogonal Mode of Control

Together, the single-particle and multibody experiments establish a direct causal chain from acid–base chemistry to optical matter architecture. Changes in pH modify the protonation state of the polyaniline shell, which alters its optical absorption. This change in absorption directly modulates the absorption-driven optical force, and the resulting force magnitude determines whether the system remains in small, disordered aggregates or evolves toward larger ordered assemblies. Although each step of this sequence might be anticipated when considered separately, the present results integrate them within a single experimentally simple platform.

This mechanism has direct implications for sensing and programmable matter, where chemical information is translated into optical, mechanical, or structural responses. In sensing, a signal encoded in observables such as particle velocity, assembly size, or degree of ordering can provide quantitative readouts of local analyte concentration, enabling a spatially resolved analytical platform. For example, a pH gradient or a localized perturbation in acidity or basicity would generate position-dependent absorption and therefore position-dependent assembly or disassembly, yielding a structural map of the chemical landscape. Moreover, while demonstrated here using changes in pH, this principle is generalizable to a broad class of chromogenic systems whose absorption properties respond to a range of chemical stimuli, including redox potential or the binding of specific analytes, and which could be used to sense the presence of specific biomolecules (e.g., amino acids or glutathione).

Beyond sensing, the same mechanism enables programmable matter when chemical stimuli are used to control assembly formation rather than merely report on it. This aligns with classical definitions of programmable matter, in which structure and function are specified by external instructions rather than by a fixed material composition. As a result, a single material can adopt different configurations, and therefore different functional properties (e.g., optical response, mechanical behavior, or electrical conductivity), in response to changes in its environment.^56^

In the present system, the chemical conditions determine how the particles interact and organize under illumination, allowing the system to switch between different assembly states. Hence, chemical gating provides a general route to chemo-adaptive optical matter, where both structure and function can be controlled. Importantly, because the underlying acid–base reaction is chemically reversible, the corresponding changes in absorption, force generation, and assembly state are also reversible, supporting repeated state switching, resetting, and reconfiguration of optical matter rather than static outcomes.

This mode of control is practically and conceptually different from simply varying the laser intensity. Increasing irradiance raises the photon flux and therefore scales the magnitude of the optical force, but it does not introduce coupling to the surrounding environment. Chemical gating, by contrast, links the optical response of the particles to the local chemical state, allowing the system to respond differently under identical illumination conditions. This distinction is particularly relevant in the context of sensing, where environmental information must be encoded into the material response. Indeed, chemistry and optics can operate as independent inputs in a combined platform, where illumination defines whether assembly is activated, and the chemical state modulates its extent or kinetics.

The temporal characteristics of chemical gating are likewise different from those of optical control. Changes in laser irradiance can be implemented essentially instantaneously on the time scale of these experiments, as modern optical modulators and drivers (e.g., acousto-optic/electro-optic devices) routinely operate with submillisecond response times. In contrast, switching the chemical environment is expected to be driven mainly by transport, mixing, diffusion, and interfacial exchange of protons, rather than by the protonation reaction itself, whose bimolecular rate approaches the diffusion limit in water (on the order of 10^10^ M^–1^ s^–1^). Because the polyaniline is localized predominantly near the particle surface, equilibration should be limited primarily by how fast the local chemical environment is exchanged. In our present study, acidic and basic conditions were prepared prior to imaging, so we do not report a measured response time for a pH jump. Nevertheless, for a representative chamber thickness of ∼100 μm, the characteristic diffusion time t ≈ L^2^/(2D) is on the order of ∼0.5–1 s for the pH-carrying ions (D_H^+^_ ≈ 9.3 × 10^–9^ and D_OH^–^_ ≈ 5.3 × 10^–9^ m^2^ s^–1^);^57^ thicker geometries would be expected to respond more slowly unless assisted by microfluidic exchange or localized chemical delivery.

On balance, chemical gating does more than modulate the strength of optical forces: it couples optical matter to its chemical environment, providing an orthogonal mode of control that can be reversible, spatially defined, and temporally different from direct optical modulation. Moreover, this strategy could be extended to other chromogenic systems, including dyes operating at different wavelengths and responsive to diverse stimuli such as pH, redox state, ion concentration, or biomolecular binding. Taken together, chemical gating establishes a new control axis for optical matter in which light determines activation, while localized chemical stimuli program structure, dynamics, and function. This separation enables adaptive, environment-aware photonic materials that cannot be achieved through optical control alone.

## 3 Conclusion

We demonstrate that chemical gating provides a fundamentally different mode of control in optical matter, in which a chemical stimulus regulates particle motion and multiparticle organization through absorption-driven optical forces without changing the illumination conditions. Using polyaniline-doped polystyrene microparticles as a proof of concept, we show that absorption-driven optical forces are reversibly modulated by pH through protonation-dependent tuning of the chromophore’s absorption. As confirmed by 3D single-particle tracking, increased absorption directly translates into enhanced optical propulsion, with particle speed following the protonation state of the chromophore. Beyond single-particle dynamics, this chemically gated control extends to multibody systems, where light-driven colloidal assemblies exhibit striking pH-dependent behavior. Under basic conditions, a stronger absorption force generates larger interfacial deformations, driving the formation of larger and more ordered colloidal assemblies, whereas reduced absorption at acidic pH restricts assembly to small, disordered clusters. Importantly, this behavior is reversible and can therefore be switched through acid–base cycling. By coupling optical forces to local chemical stimuli, chemical gating offers an orthogonal and powerful complement to beam engineering, filling a conceptual gap between beam-defined optical architectures and chemically adaptive materials. This integration enables chemical inputs to be converted into mechanical motion or structural organization via light, extending optical matter toward sensing and programmable systems. Together, these insights establish a versatile framework for chemically controlling light-matter interactions and provide new design principles for dynamic, adaptive, and responsive photonic materials.

## 4 Experimental Section

### 4.1 Synthesis and Characterization of PDPS MPs

#### 4.1.1 Synthesis of Polystyrene Microparticles (PS MPs)

PS MPs were synthesized via free radical dispersion polymerization in ethanol using PVP as a stabilizer, modifying the first step of the protocol described by Chen et al.^48^ To form the PS MPs, 1 g of polyvinylpyrrolidone (PVP-40, Sigma-Aldrich) was dissolved in 100 mL of ethanol (Sigma-Aldrich). The resulting solution was transferred to a three-neck flask. Then, 25 mL of styrene (≥99%, Sigma-Aldrich) and 0.2 g of azobis­(isobutyronitrile) (AIBN, ≥98%, Sigma-Aldrich) were added to the flask solution under a nitrogen atmosphere. The reaction mixture was heated at 75 °C and refluxed overnight at 300 rpm. After cooling, the solution was centrifuged at 2254 g for 5 min and washed with ethanol five times. The final particle suspension was stored at room temperature.

#### 4.1.2 Synthesis of Polyaniline-Doped Polystyrene Microparticles (PDPS MPs)

To functionalize the surface of the PS MPs, aniline was polymerized using a swelling synthesis method with an aniline-to-ammonium persulfate ratio of 1:5. PS MPs (0.4 g) were dispersed in a solution consisting of 800 μL of 0.5 M hydrochloric acid (HCl, 37%, Sigma-Aldrich), 0.5 μL of aniline (Sigma-Aldrich), and 310 μL of a 20 mg/mL ammonium persulfate (APS, ≥98%, Sigma-Aldrich) solution. The mixture was stirred at room temperature overnight and then washed by centrifugation at 7379 g for 5 min, three times with ethanol and three times with water.

#### 4.1.3 Characterization of the Microparticles (MPs)

i) Particle size distribution: PS MPs and PDPS MPs were characterized using a Mastersizer 3000 (Malvern Instruments Ltd.). For the PS MPs samples, the sample was considered to be spherical, the polystyrene refractive index set at 1.57 and the absorbance at 0.01. H_2_O was used as dispersant (refractive index of 1.334). The sample was measured at a laser obscuration of 3.15% with 10 replicate measurements. For the PDPS MPs samples, the sample was also considered to be spherical, polyaniline’s refractive index set at 1.59, and the absorbance at 0.01. H_2_O was again used as dispersant. The sample was measured at 4.33% laser obscuration with 10 replicate measurements.
ii) Evolution of size distribution at different pH: Measurements were performed using a Zetasizer Nano ZS (Malvern Instruments Ltd.) equipped with a 4 mW He–Ne red light laser using a laser wavelength of 633 nm. The detector is placed at angle of 173° to the sample. Hydrodynamic diameters were determined with a sample diluted 1:100 in Milli-Q (200 μL) in 1 cm × 1 cm polystyrene cuvette. The measurement was performed at 25 °C with 30 s of equilibrium time. The measurements were performed in triplicate with 10 runs per measurement. Data was analyzed by cumulant and CONTIN analysis to obtain the hydrodynamic size.
iii) Morphology characterization: The morphology of the samples was observed by scanning electron microscopy (SEM, JSM-7600F, JEOL). The sample was gold-coated under vacuum.
iv) Absorption spectra: Measurements were recorded using a Cary 6000i UV–vis-NIR spectrophotometer equipped with an integrating sphere (Varian DRA 2500 00–100811–00) to account for the light scattering from the MP suspension. The absorption spectra were measured in 1 cm quartz cuvettes. The PDPS MPs were added to an acidic (pH 2, HCl) and a basic (pH 10, NaOH) solution to a final concentration of 2% w/v.

### 4.2 Sample Preparation

#### 4.2.1 Single-Particle System

The stock suspension of particles was first sonicated and then diluted 1:20 with Milli-Q water. To set the pH, a series of hydrochloric acid (HCl, 37%, Sigma-Aldrich) and sodium hydroxide (NaOH, pellets, Sigma-Aldrich) solutions spanning pH 1–11 were prepared, and 117 μL of the selected pH solution was mixed with 3 μL of the diluted particle suspension. This gave a final particle dilution of 1:40 (1:800 relative to the original stock) while maintaining the intended pH. The mixtures were then vortexed and sonicated to ensure uniform dispersion. Experiments at different irradiances were performed using a pH 10 suspension.

Circular glass coverslips (35 mm diameter) were cleaned and treated with UV-Ozone for 60 min to enhance surface hydrophilicity. A Grace Bio-Laboratories imaging spacer (20 mm diameter, 0.12 mm thickness) was glued to each treated coverslip, and 8 μL of the prepared particle suspension was added to the chamber. The chamber was sealed with a clean coverslip to prevent evaporation during imaging.

#### 4.2.2 Multibody System

The stock suspension of particles was first sonicated and then diluted 1:100 in deuterium oxide (D_2_O, 99.9% atom, Sigma-Aldrich). To adjust the pH, 5 μL of either 0.1 M HCl or 0.001 M NaOH was added to 45 μL of the diluted particle suspension. This step yielded final pH values of approximately 2 (acidic) and 10 (basic), as confirmed with a pH-meter. The resulting mixtures were then vortexed and sonicated to ensure homogeneity. For the intermediate pH experiments, samples at pH 1, pH 3, and pH 5 were prepared in the same way, using 5 μL of 1 M HCl, 5 μL of 10^–2^ M HCl, or 5 μL of 10^–4^ M HCl, respectively. The reported pH values correspond to uncorrected glass-electrode readings obtained in D_2_O (pD ≈ pH + 0.4).

The pH-cycling experiments were performed by alternately adding 1 M HCl or 1 M NaOH to 990 μL of the diluted particle suspension, switching the sample between pH 2 and pH 10. After each addition, the suspension was briefly mixed and an aliquot was withdrawn for imaging (Table 1). This procedure minimized sample dilution throughout the pH-cycling process. Successful switching between the emeraldine salt and base states was further confirmed visually from the color change of the suspension.

**Table 1:** Sequential pH-Cycling Protocol, Alternating between Acidic (pH 2) and Basic (pH 10) Conditions

Step	Start	Volume added	Volume removed	Sample
1	990.0 μL at pH 7	10.0 μL of 1 M HCl	10.0 μL	A1 (pH 2)
2	990.0 μL at pH 2	10.0 μL of 1 M NaOH	10.0 μL	B2 (pH 10)
3	990.0 μL at pH 10	10.1 μL of 1 M HCl	10.1 μL	A3 (pH 2)
4	990.0 μL at pH 2	10.0 μL of 1 M NaOH	10.0 μL	B4 (pH 10)
5	990.0 μL at pH 10	10.1 μL of 1 M HCl	10.1 μL	A5 (pH 2)
6	990.0 μL at pH 2	10.0 μL of 1 M NaOH	10.0 μL	B6 (pH 10)

Rectangular glass coverslips (22 × 40 mm) were cleaned in a 10% Hellmanex III detergent solution (Hellma). The coverslips were submerged in the solution, sonicated for 10 min, and left in the detergent solution overnight. Afterward, the coverslips were transferred to a Milli-Q water bath and sonicated for 10 min, repeated three times with new water each time. In order to have a D_2_O/air interface within the sample chamber, a glass ring (1.5 cm thickness) was glued onto the cleaned coverslip, and 10 μL of the prepared particle suspension was added to the chamber. Finally, a second coverslip was glued onto the glass ring to seal the chamber.

### 4.3 Imaging

#### 4.3.1 Single-Particle System

Experiments were performed using a home-built multiplane (3D) microscope (Figure S1) with a 60x air objective (UPFLN NA 0.90, Olympus). Movies were recorded under dark-field illumination from a halogen lamp coupled to an oil-immersion dark-field condenser (NA 1.4, Olympus). A 561 nm diode-pumped solid-state laser (Sapphire 100 mW, Coherent Inc.) was also directed through the objective to generate homogeneous widefield excitation (447 W/cm^2^), used to induce absorption forces on the particles. Three-dimensional imaging was achieved using a prism-based multiplane module (Figure S1) that splits the signal into eight depth planes, detected simultaneously on two sCMOS cameras (Orca Flash 4.0, Hamamatsu). This enabled multiplane acquisition without mechanical scanning over a total volume of 50 × 50 × 4 μm^3^. The two cameras were synchronized using a National Instruments board (USB-6343, National Instruments) and a custom LabVIEW software, with a 100 ms exposure time for each frame. For the experiments at different irradiances, the laser power was adjusted using the laser control unit and neutral density filters, and measured with a power meter (PM100D, S130C photodiode power sensor, Thorlabs). The beam area was determined using a CMOS beam profiler (WinCamD-LCM, DataRay).

#### 4.3.2 Multibody System

Brightfield movies were recorded on an inverted microscope (IX71, Olympus) with a 40x water-immersion objective (NA 1.2, Zeiss). Samples were illuminated with a halogen lamp, and images were collected by an EM-CCD camera (ImagEM Enhanced, Hamamatsu Photonics) with an exposure time of 31 ms per frame. Absorption forces were induced with a 561 nm diode-pumped solid-state laser (Sapphire 200 mW, Coherent Inc.) under widefield excitation (114 W/cm^2^).

To assess reversibility under repeated irradiation, the sample was subjected to alternating periods of 15 s irradiation and 30 s recovery, controlled by opening and closing a shutter while the laser remained continuously on. For experiments performed at different irradiances, the laser power was adjusted and measured as described above for the single-particle system.

### 4.4 Analysis

#### 4.4.1 Single-Particle System

Multiplane microscopy was calibrated following the protocol described in Louis et al.^58^ Single-particle tracking was performed using a customized algorithm, adapted from Louis et al.,^58^ but tailored to the larger MPs and dark-field contrast. The pipeline comprised the following steps: (1) Otsu’s thresholding and centroid detection in each plane; (2) cross-plane grouping of the same particle; (3) axial (z-) position estimation from focus-dependent image gradient fits; and (4) frame-to-frame linking and tracking within a radius threshold. MSDs and particle speeds were calculated from the resulting trajectories. For PDPS data sets, a z-filter removed very short z-traces to stabilize MSD and speed calculations. See Supporting Information for further details.

#### 4.4.2 Multibody System

Image analysis was performed using Fiji/ImageJ software. For quantitative analysis, five independent movies were analyzed for each condition, with each movie containing at least 126 MPs. A frame acquired after 15 s of laser irradiation was selected for each movie. Particles were manually counted and classified into different assembly sizes based on proximity; assemblies were defined as particles in direct contact.

## Supplementary Material















## Notes

ESI_Chemical modulation of optical forces for dynamic light-driven materials: includes further experimental details, an estimation of the heat release and temperature elevation under widefield excitation, and supporting figures, including controls, SPT data, and a schematic representation of the experimental setup of the multiplane microscope (PDF)
Movie S1: movie played in real time showing the dynamic assembly behavior of PDPS MPs under basic conditions (scale bar = 20 μm). This movie corresponds to the representative frames shown in Figure 4 (MP4)
Movie S2: movie played in real time showing the dynamic assembly behavior of PDPS MPs under basic conditions (scale bar = 20 μm) (MP4)
Movie S3: movie played in real time showing the dynamic assembly behavior of PDPS MPs under acidic conditions (scale bar = 20 μm). This movie corresponds to the representative frames shown in Figure 4 (MP4)
Movie S4: movie played in real time showing the dynamic assembly behavior of PDPS MPs under acidic conditions (scale bar = 20 μm) (MP4)
Movie S5: movie played at 3x speed showing the reversible assembly behavior of PDPS MPs under basic conditions across successive laser on/off cycles (scale bar = 20 μm). This movie corresponds to the representative frames shown in Figure S9 (MP4)
Movie S6: composite of six movies played in real time showing the reversible assembly behavior of PDPS MPs across successive pH cycles (scale bar = 20 μm). “A” denotes an acidic state and “B” a basic state, while the numbers indicate the sequence of the transitions. This movie corresponds to the representative frames shown in Figure S10 (MP4)
