# Supplementary material for: Chemical Modulation of Optical Forces for Dynamic Light-Driven Materials
Source: ACS Photonics. 2026 Jul 31;13(16):4577–87. doi: 10.1021/acsphotonics.6c00535 (PMC13501513; doi:10.1021/acsphotonics.6c00535)
Supplement: Supplementary file 1 [file ph6c00535_si_001.pdf]

ELECTRONIC SUPPLEMENTARY INFORMATION:

Chemical modulation of optical forces for dynamic light-driven materials

Laia Torrella-Adriaensen,[†‡] Boris Louis,[‡] Jagannath Satpathy,[‡] Anna Tarrats-Duran,[†] Jose Rodrigo Magaña,[†] Cristina Fornaguera,[†] Santi Nonell,[†] Susana Rocha,[‡] Johan Hofkens,[‡] Roger Bresolí-Obach[*†]

† Institut Químic de Sarrià, Universitat Ramon Llull, Via Augusta 390, 08017 Barcelona, Spain

‡ Department of Chemistry, Katholieke Universiteit Leuven, Celestijnenlaan 200F, 3001 Heverlee, Belgium

* roger.bresoli@iqs.url.edu

TABLE OF CONTENTS

1. Single-particle system analysis 3

2. Estimation of the heat release and temperature elevation under widefield excitation 5

3. Supporting figures 7

4. References 16

1. SINGLE-PARTICLE SYSTEM ANALYSIS

Calibration of the multiplane microscope and the corresponding data was performed according to Louis et al.,[1] and single-particle tracking was performed using a custom-written algorithm, adapted from Louis et al.[1] The analysis code was adapted to account for the larger particle size and the more complex three-dimensional intensity profiles of the polystyrene (PS) spheres in dark-field imaging mode. Despite these modifications, the overall processing workflow followed the same general structure: (1) particle detection in all planes and frames, (2) consolidation of particle localization across planes, (3) axial position calculations, and (4) spatiotemporal tracking.

For particle detection, each plane was independently thresholded using Otsu's method[2], after which the centroid of each detected object was calculated. Localizations corresponding to the same physical particle in different planes were grouped based on a distance-based criterion. Given the particle diameter of approximately 20 pixels, localizations separated by only a few pixels across adjacent planes were assigned to the same particle. Axial position determination was based on focus-dependent image gradients. For each particle, a region of interest (ROI) of ± 15 pixels around the centroid was extracted in all planes, and the mean image gradient within this ROI was computed. The plane exhibiting the maximum gradient corresponds to the in-focus position. The dependence of the image gradient on the axial (z-) position was subsequently fitted to determine the particle's axial coordinate. After determining the full three-dimensional particle coordinates (x-, y-, z-) for each frame, particle positions were linked over time to form trajectories. Multiplane connections were performed using a radius threshold, chosen such that a particle is not expected to move beyond this distance between consecutive frames.

Additionally, for this work, a z-filter was applied as a quality-control step to exclude traces that were too short to reliably track them along the z-axis, thereby reducing short-track artifacts in MSD and speed calculations. In the experimental datasets (PDPS MPs), particles showed clear and consistent upward motion along the z-axis (Figure S2). Therefore, short z-trajectories were excluded, as they most likely correspond to particles that were present in the imaging volume for only a limited number of frames (e.g., entering or leaving the focal region) and were thus too short for reliable MSD analysis.

In contrast, control particles (PS MPs) moved very slowly and did not show directed z-motion, resulting in intrinsically short traces. Therefore, applying the same filter would have artificially removed valid control data.

Consistent with this behavior, applying the z-filter to the experimental datasets (PDPS MPs) retained most traces across all pH conditions, with 66% to 90% of the traces remaining (89% at pH 10 and 66% at pH 2). In comparison, fewer than 20% of control (PS MPs) traces (10% at pH 10 and 20% at pH 2) would have met the same criterion, consistent with the absence of directed z-motion in the controls.

Altogether, these results show that the traces excluded from the experimental datasets were too short to allow reliable analysis, rather than representing a distinct particle behavior. This supports the use of the z-filter for the experimental samples (PDPS MPs), where it improves trace quality while retaining a representative fraction of particles, and justifies its exclusion for the control samples (PS MPs). For reference, unfiltered results (Figure S3) show the same overall trends (speed as a function of pH) as the filtered data presented in Figure 3B, indicating that the z-filter

does not bias the conclusions. Representative filtered and unfiltered traces are shown in Figure S2, illustrating the overall upward motion and the short traces removed by the z-filter.

MSD analysis was used to characterize particle motion. MSDs were calculated from individual particle trajectories as the average squared displacement as a function of lag time, separately for lateral (x-, y-) and axial (z-) motion. The resulting MSD curves were averaged over all particles, and the associated standard deviations were used to estimate the variability across trajectories.

Particle speeds were calculated from tracked trajectories as frame-to-frame displacements per unit time. For each particle, positional differences between consecutive frames were computed along the x-, y-, and z-directions, averaged over the trajectory, and converted from nanometers per frame to micrometers per second using the frame interval. These values represent the average instantaneous particle speed.

Both the localization and tracking algorithms are freely available in its original version (https://github.com/BorisLouis/3D-image-processing) and the adapted version developed for this work (https://github.com/BorisLouis/AbsorptionForce_AxialTracking).

2. ESTIMATION OF THE HEAT RELEASE AND TEMPERATURE ELEVATION UNDER WIDEFIELD EXCITATION

We can estimate the frequency of excitation events (f_{Ex}; $S_0 \rightarrow S_1$) for a single dye molecule using Eq. S1. s_D, $I_{Ex,561}$ and $E_{Ex,561}$ are the dye's absorption cross section, the 561 nm laser irradiance and the energy of a single 561 nm photon, respectively. As the molar absorption coefficient (e) of the dye is unknown, we assumed an *e* value of 5×10^4 L/(mol·cm^{-1}), which is a realistic value for common chromophores. From this *e* value, we can calculate the *s* as 1.9×10^{-20} m^2.

Single particle system

$$f_{Ex,D} = \frac{\sigma_D \cdot I_{Ex,561}}{E_{Ex,561}} = \frac{(1.9 \cdot 10^{-20}\, m^2) \cdot (4.5 \cdot 10^6\, W \cdot m^{-2})}{(3.54 \cdot 10^{-19}\, J)} = 2.4 \cdot 10^5\, s^{-1} \qquad \boldsymbol{Eq. S1}$$

Multibody system

$$f_{Ex,D} = \frac{\sigma_D \cdot I_{Ex,561}}{E_{Ex,561}} = \frac{(1.9 \cdot 10^{-20}\, m^2) \cdot (1.1 \cdot 10^6\, W \cdot m^{-2})}{(3.54 \cdot 10^{-19}\, J)} = 0.59 \cdot 10^5\, s^{-1} \qquad \boldsymbol{Eq. S1'}$$

The polyaniline chromophore is non-emissive; therefore, all absorbed photon energy is dissipated as heat. Consequently, the heat power released by each dye molecule (Q_D) is calculated using Eq. S2.

Single particle system

$$Q_D = f_{Ex,D} \cdot E_{Ex,561} = (2.4 \cdot 10^5\, s^{-1}) \cdot (3.54 \cdot 10^{-19}\, J) = 8.5 \cdot 10^{-14}\, W \qquad \boldsymbol{Eq. S2}$$

Multibody system

$$Q_D = f_{Ex;D} \cdot E_{Ex,561} = (0.59 \cdot 10^5\, s^{-1}) \cdot (3.54 \cdot 10^{-19}\, J) = 2.1 \cdot 10^{-14}\, W \qquad \boldsymbol{Eq. S2'}$$

The total heat power released per PS MP (Q_{MP}) is calculated by summing the Q_D generated by all dye molecules (n_D). According to common manufacturers (e.g. ThermoFisher), the maximum dye loading for MPs of this size is approximately 3.1×10^7 equivalents of fluorescein.[3] We assume that the number of dye molecules per particle is equal to the fluorescein equivalents. This assumption provides an upper-bound estimate of heat generation and therefore represents a conservative scenario. Using this value, Q_{MP} is calculated according to Eq. S3.

Single particle system

$$Q_{MP} = n_D \cdot Q_D = (3.1 \cdot 10^7) \cdot (8.5 \cdot 10^{-14}\, W) = 2.6 \cdot 10^{-6}\, W \qquad \boldsymbol{Eq. S3}$$

Multibody system

$$Q_{MP} = n_D \cdot Q_D = (3.1 \cdot 10^{7}) \cdot (2.1 \cdot 10^{-14}\, W) = 6.5 \cdot 10^{-7}\, W \qquad \boldsymbol{Eq. S3'}$$

We can calculate the temperature elevation (*T(r)*) of a single isolated MP according to steady-state heat transfer equation for a sphere (Eq. S4), where λ is the thermal conductivity of water at 298 K (0.60 W/m·K) and *r* is the radial distance from the center of the MP.

$$\frac{dT(r)}{dr} = -\frac{Q_{MP}}{4 \cdot \pi \cdot \lambda} \cdot \frac{1}{r^2} \qquad \boldsymbol{Eq. S4}$$

The equations for the temperature increase inside the MP (DT_o; Eq. S5) and the heat release outside the MP (Eq. S6) are derived by integrating Eq. S4, assuming that the temperature inside the MP (T_o) is homogeneous under steady-state approximation, where r_o is the radius of the MP.

Single particle system

$$(0 \le r \le r_o)\ \Delta T(r) = \Delta T_o = \frac{Q_{MP}}{4 \cdot \pi \cdot \lambda} \cdot \frac{1}{r_o} = \frac{(2.6 \cdot 10^{-6}\, W)}{4 \cdot \pi \cdot \left(0.60 \frac{W}{m} \cdot K\right)} \cdot \frac{1}{1 \cdot 10^{-6}\, m} = 0.3\, K\ \boldsymbol{Eq. S5}$$

Multibody system

$$(0 \le r \le r_o)\ \Delta T(r) = \Delta T_o = \frac{Q_{MP}}{4 \cdot \pi \cdot \lambda} \cdot \frac{1}{r_o} = \frac{(6.5 \cdot 10^{-7}\, W)}{4 \cdot \pi \cdot \left(0.60 \frac{W}{m} \cdot K\right)} \cdot \frac{1}{1 \cdot 10^{-6}\, m} = 0.09\, K\ \boldsymbol{Eq. S5'}$$

$$\Delta T(r) = \frac{Q_{MP}}{4 \cdot \pi \cdot \lambda} \cdot \frac{1}{r} \quad (r_o < r) \qquad \boldsymbol{Eq. S6}$$

3. SUPPORTING FIGURES

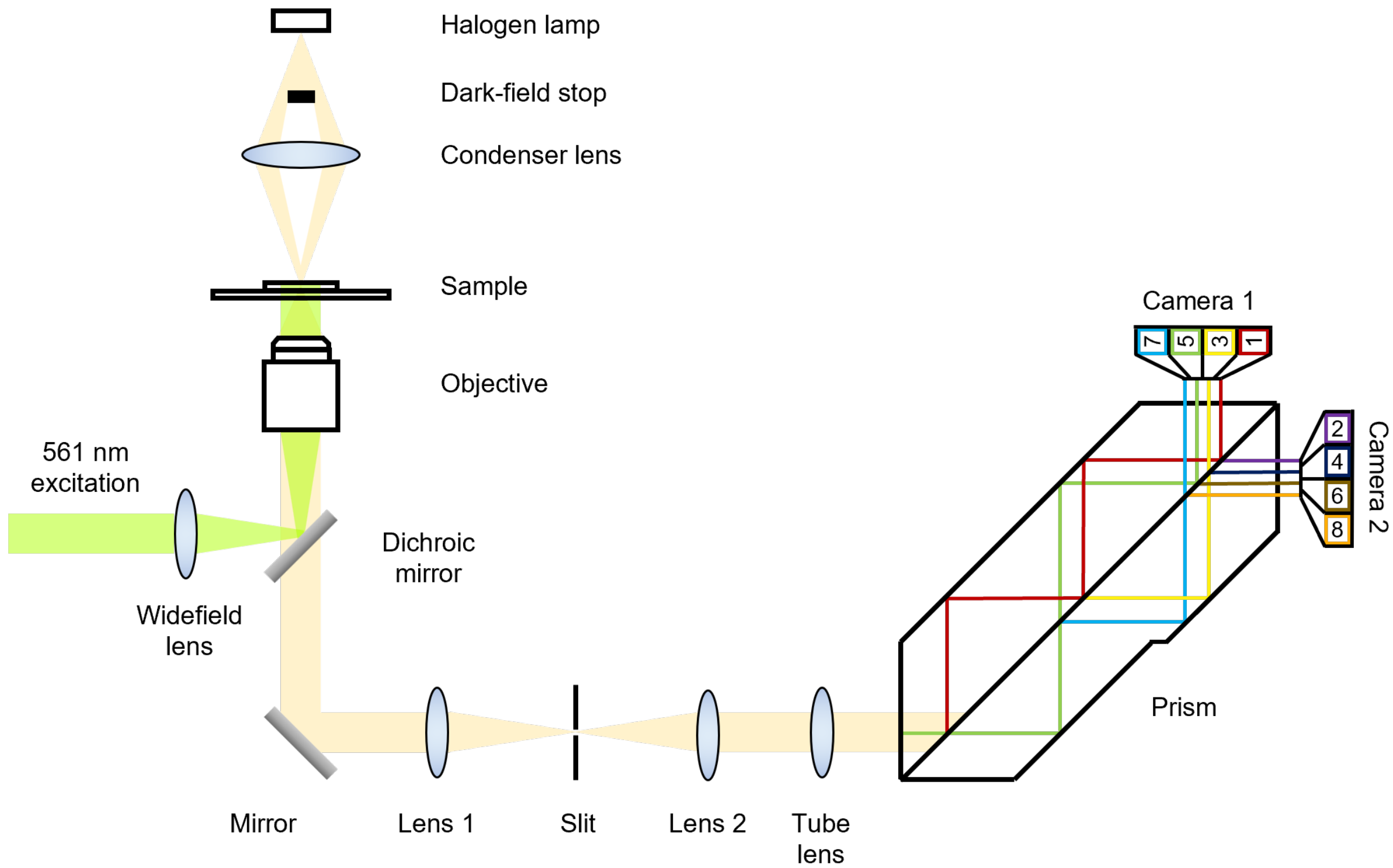

Figure S1. Optical scheme of the home-built multiplane (3D) microscope, including laser used to induce absorption forces on the sample. Each imaging plane is identified by the numbering on the two cameras.

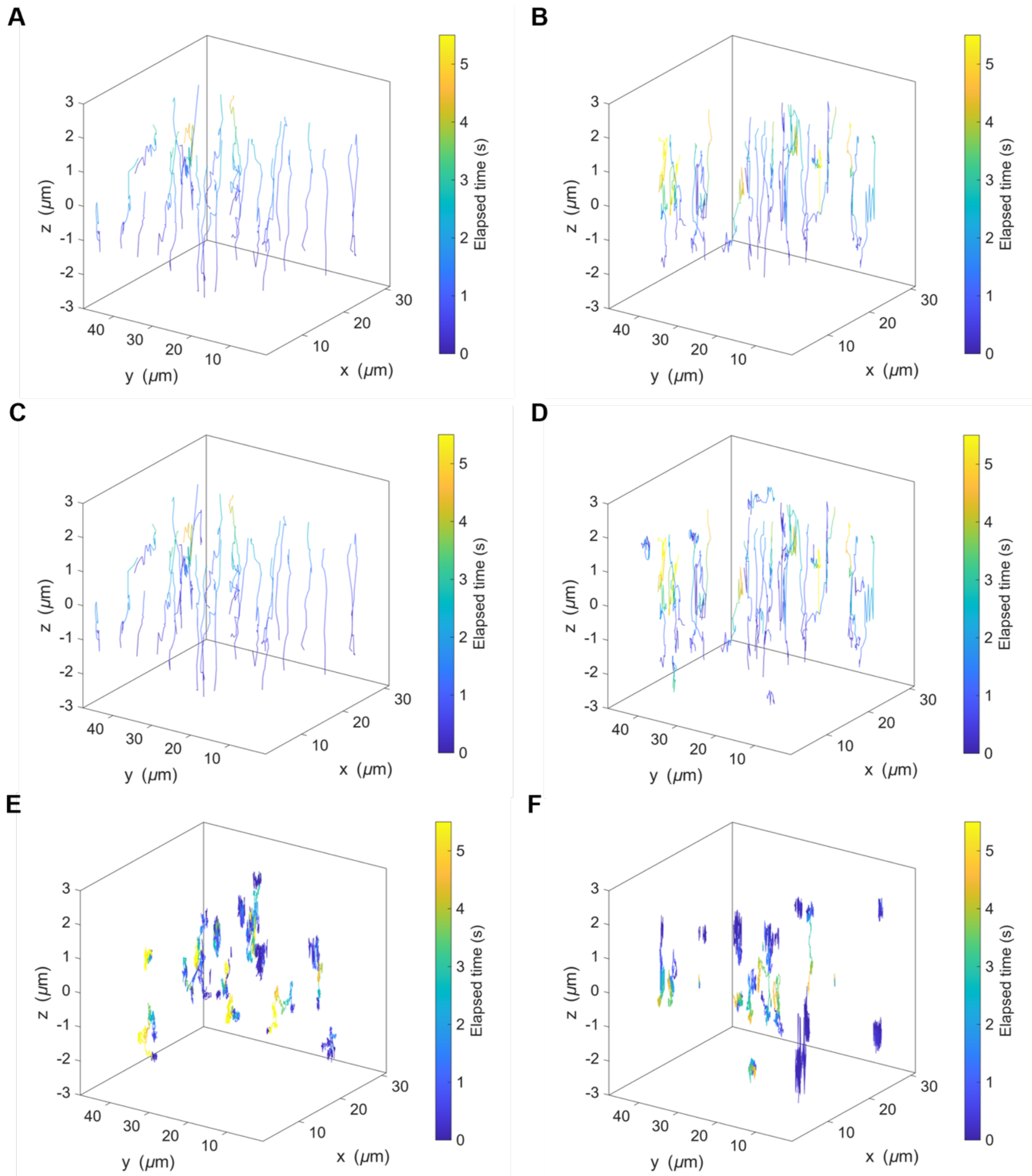

Figure S2. Representative traces obtained with the single-particle tracking algorithm for PDPS MPs (A–D) and PS MPs used as controls (E, F). The differences between traces from the filtered (A, B) and unfiltered (C, D) algorithms, in which some artifact traces with no clear motion due to the localization of the samples out of the imaging volume, motivated our choice to use the filtered analysis. (A) PDPS MPs at pH 10 (filtered). (B) PDPS MPs at pH 2 (filtered). (C) PDPS MPs at pH 10 (unfiltered). (D) PDPS MPs at pH 2 (unfiltered). (E) PS MPs (control) at pH 10 (unfiltered). (F) PS MPs (control) at pH 2 (unfiltered).

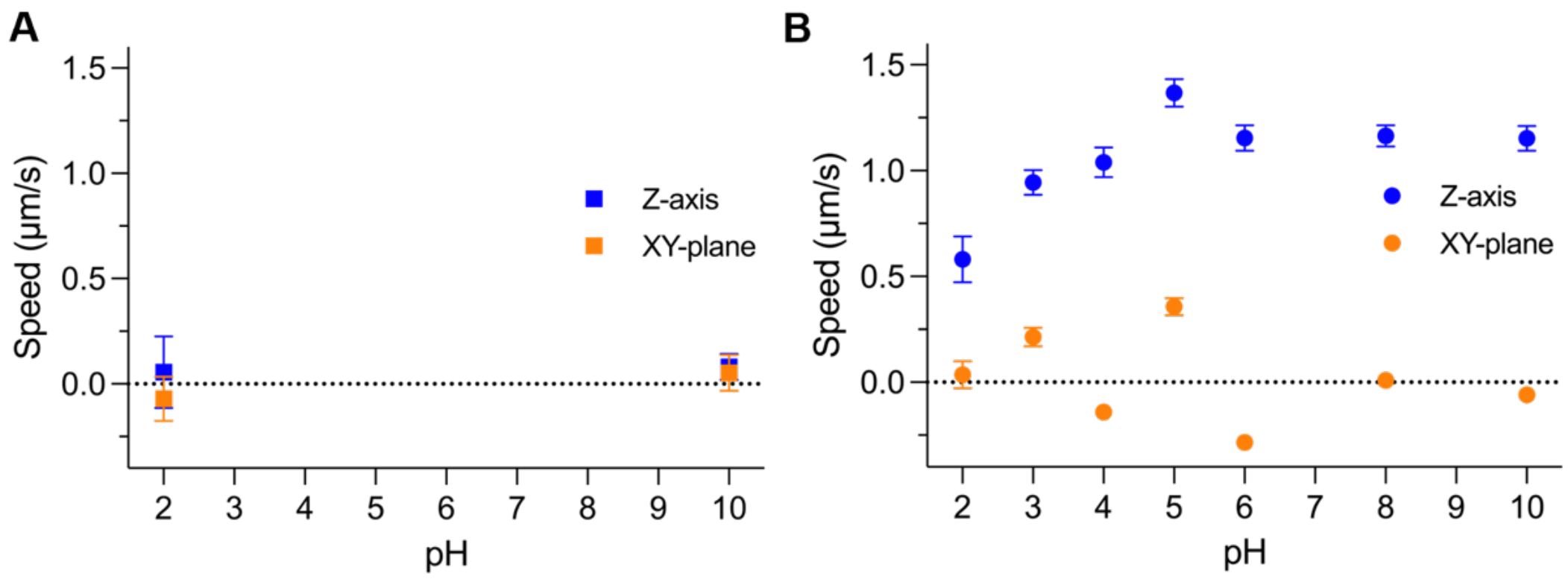

Figure S3. Speed of PS MPs and PDPS MPs along the z-direction (blue) and XY-plane (orange) as a function of pH. (A) Speed of PS MPs (control) at pH 2 (acidic pH) and pH 10 (basic pH). (B) Speed of PDPS MPs as a function of pH. In both panels, the error bars represent the standard error of the mean (SEM) and the speeds are calculated using the unfiltered algorithm.

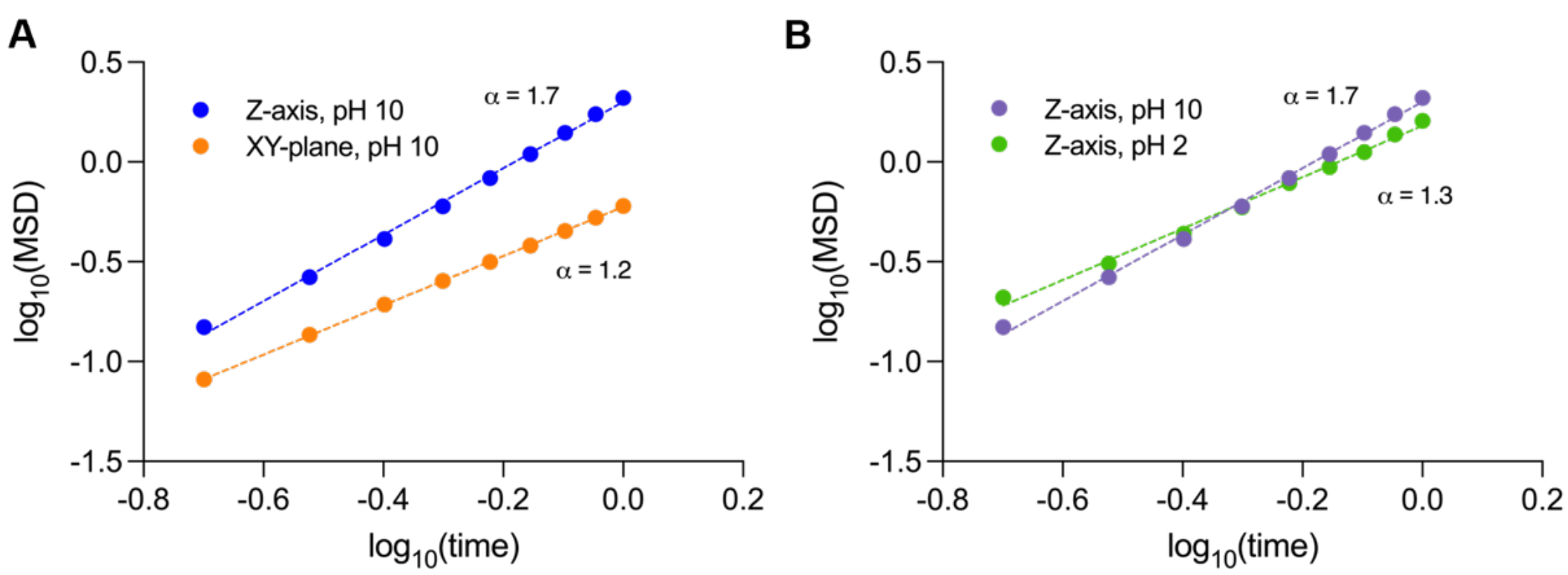

Figure S4. Mean squared displacement (MSD) analysis of PDPS MPs. (A) MSD at pH 10 along the Z-direction (blue) and XY-plane (orange) as a function of time, shown on a logarithmic scale. (B) MSD along the z-direction measured at pH 10 (purple) and pH 2 (green) as a function of time, shown on a logarithmic scale. In both (A) and (B), the slope corresponds to α, which matches the exponent in the power-law fit in Figure 3.

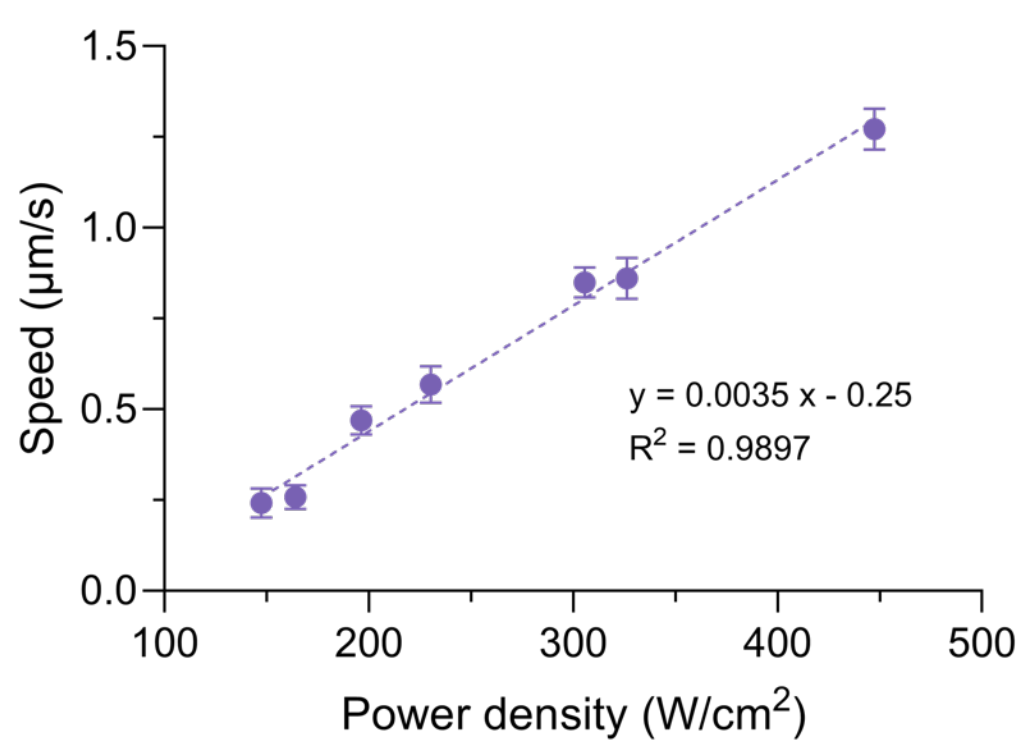

Figure S5. Speed of PDPS MPs at basic conditions along the Z-direction as a function of irradiance. The error bars represent the standard error of the mean (SEM). Speeds were calculated using the filtered algorithm. The dashed line is a linear fit, with the equation and goodness of fit (R^2) indicated in the figure.

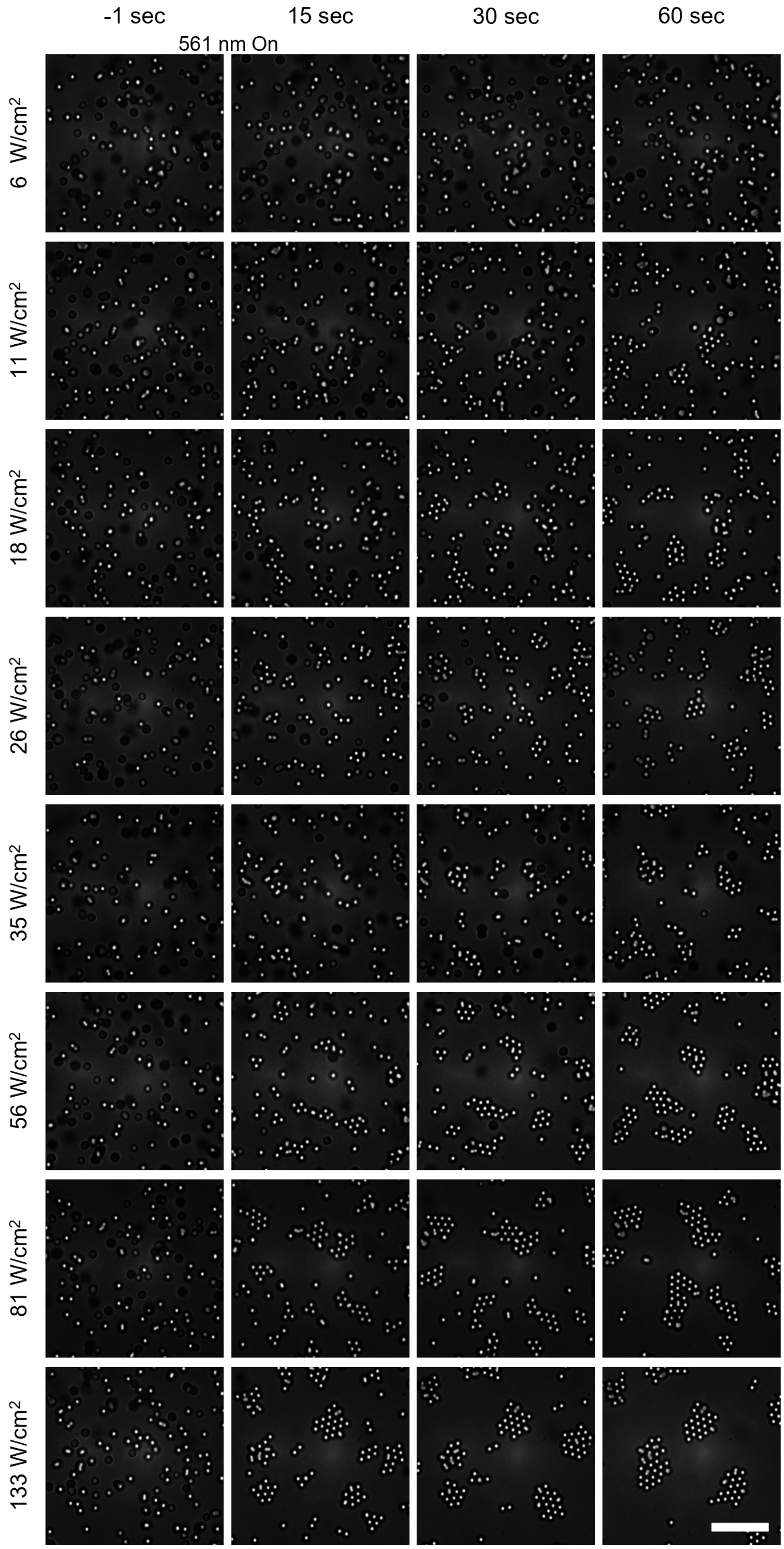

Figure S6. Time-lapse brightfield microscopy images showing the assembly behavior of PDPS MPs under basic conditions (pH 10) at different irradiances. Images are captured before (-1 sec) and after (15, 30, and 60 sec) switching on the 561 nm laser (scale bar = 20 μm).

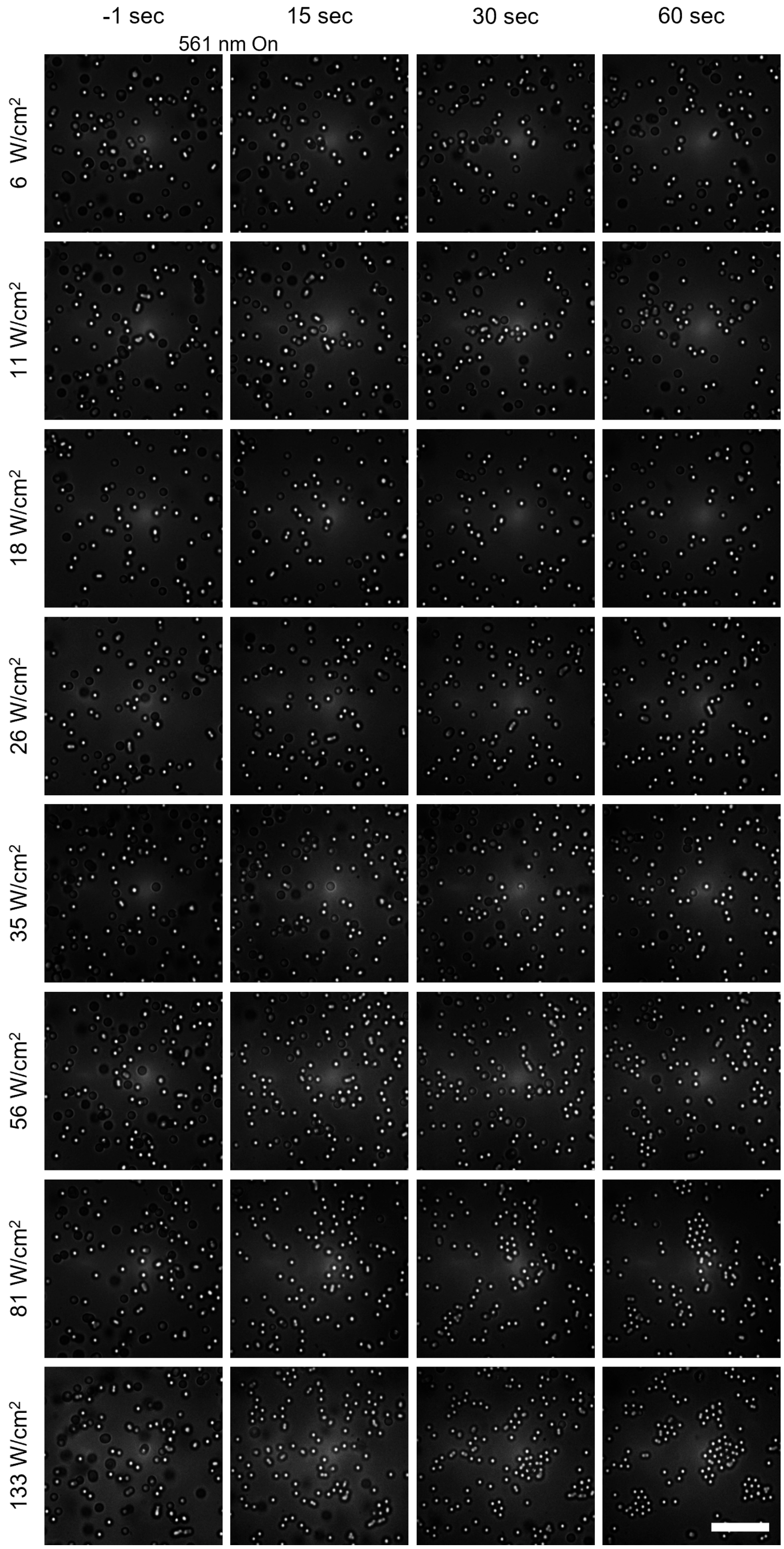

Figure S7. Time-lapse brightfield microscopy images showing the assembly behavior of PDPS MPs under acidic conditions (pH 2) at different irradiances. Images are captured before (-1 sec) and after (15, 30, and 60 sec) switching on the 561 nm laser (scale bar = 20 μm).

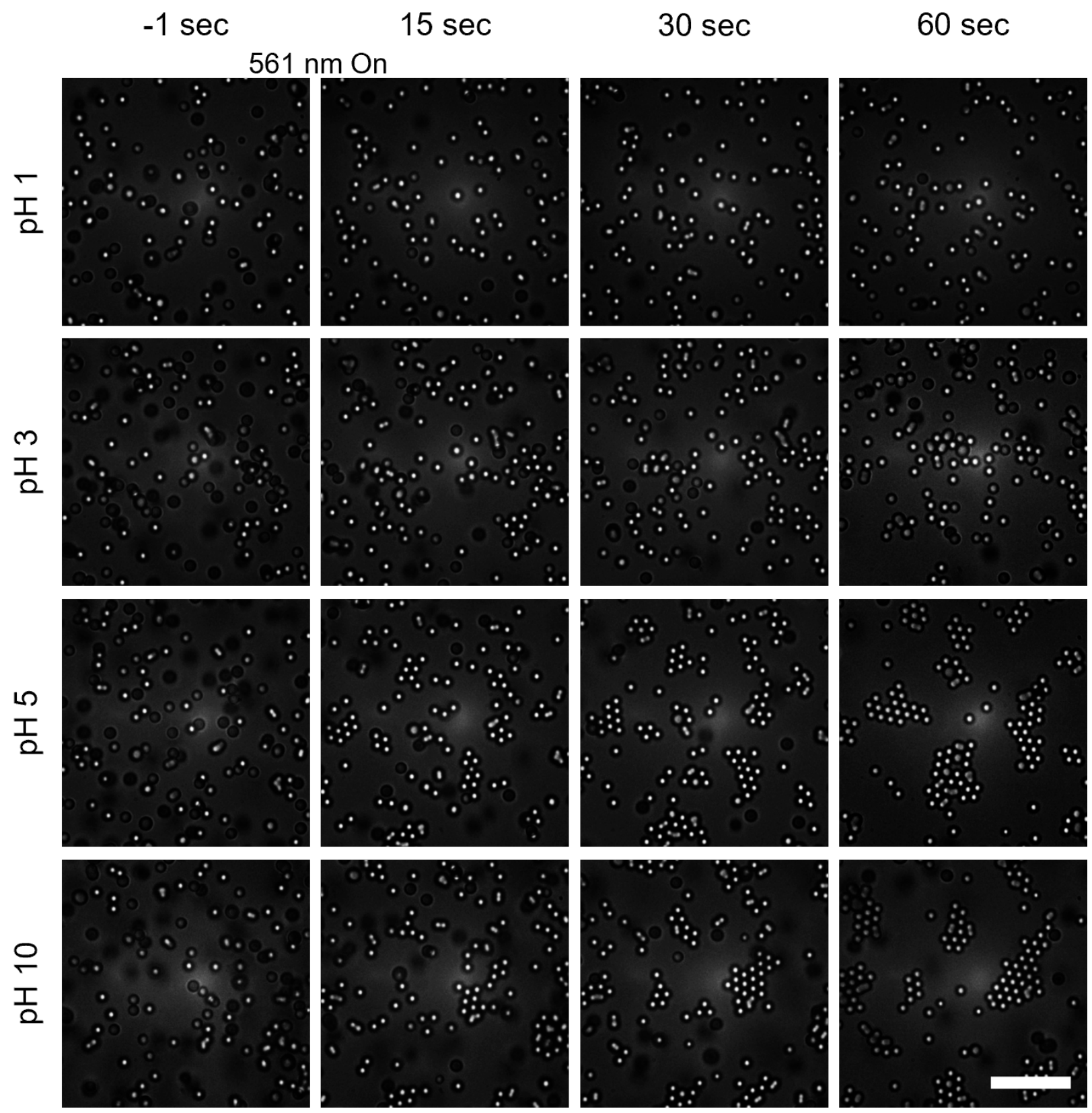

Figure S8. Time-lapse brightfield microscopy images showing the assembly behavior of PDPS MPs across the protonation transition: conditions below the pK_a (pH 1), at the pK_a (intermediate, pH 3), and above the pK_a (pH 5 and pH 10). Images are captured before (-1 sec) and after (15, 30, and 60 sec) switching on the 561 nm laser at 30 W/cm^2 (scale bar = 20 µm).

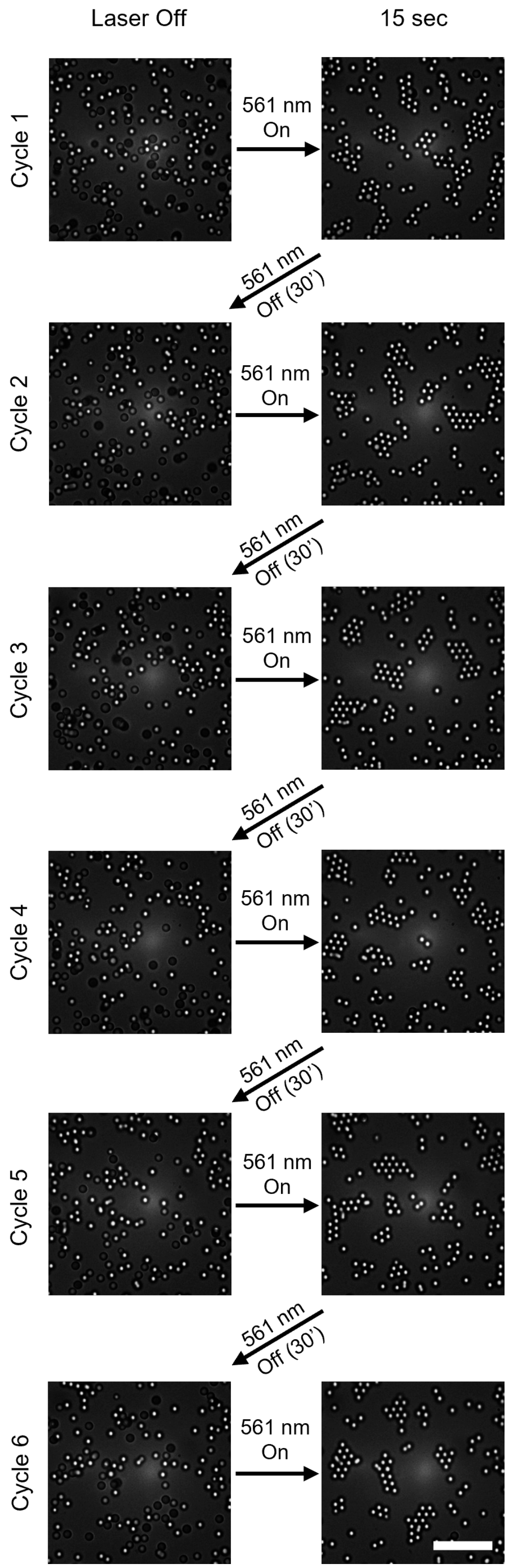

Figure S9. Time-lapse brightfield microscopy images showing the reversible assembly behavior of PDPS MPs under basic conditions (pH 10) across successive laser on/off cycles. Images are captured 15 sec after switching on the 561 nm laser at 30 W/cm^2 ("15 sec") and 30 sec after switching it off ("Laser Off") (scale bar = 20 μm).

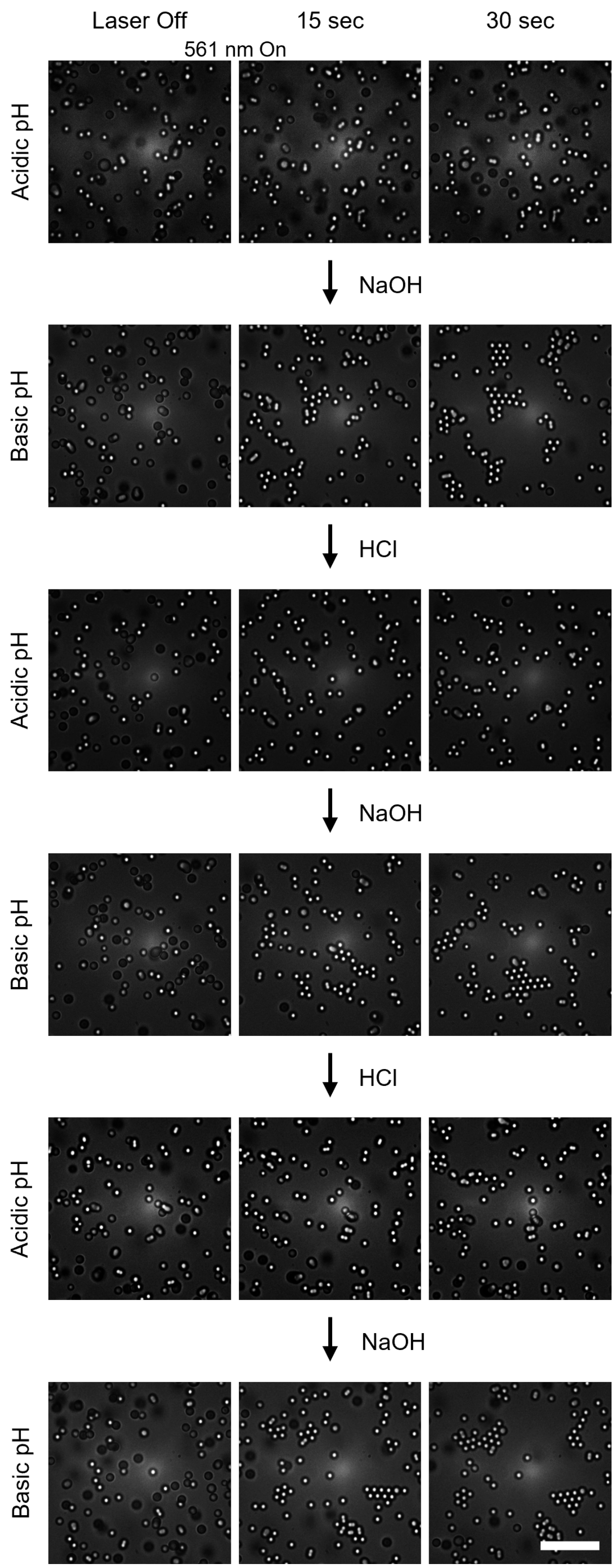

Figure S10. Time-lapse brightfield microscopy images showing the reversible assembly behavior of PDPS MPs across successive pH cycles. Images are captured before (-1 sec) and after (15, 30, and 60 sec) switching on the 561 nm laser at 30 W/cm^2 for each sample (scale bar = 20 μm). The pH was sequentially

adjusted between acidic (pH 2) and basic (pH 10) conditions using HCl and NaOH, respectively, during sample preparation, and aliquots were then extracted for imaging.

Movie S1. Movie played in real time showing the dynamic assembly behavior of PDPS MPs under basic conditions (scale bar = 20 μm). Note that minor mechanical drift is observed during the laser on/off transitions due to the operation of opening/closing the shutter. This movie corresponds to the representative frames shown in Figure 4.

Movie S2. Movie played in real time showing the dynamic assembly behavior of PDPS MPs under basic conditions (scale bar = 20 μm).

Movie S3. Movie played in real time showing the dynamic assembly behavior of PDPS MPs under acidic conditions (scale bar = 20 μm). This movie corresponds to the representative frames shown in Figure 4.

Movie S4. Movie played in real time showing the dynamic assembly behavior of PDPS MPs under acidic conditions (scale bar = 20 μm).

Movie S5. Movie played at 3x speed showing the reversible assembly behavior of PDPS MPs under basic conditions across successive laser on/off cycles (scale bar = 20 μm). This movie corresponds to the representative frames shown in Figure S9.

Movie S6. Composite of six movies played in real time showing the reversible assembly behavior of PDPS MPs across successive pH cycles (scale bar = 20 μm). "A" denotes an acidic state and "B" a basic state, while the numbers indicate the sequence of the transitions. This movie corresponds to the representative frames shown in Figure S10.

4. REFERENCES

(1) Louis, B.; Camacho, R.; Bresolí-Obach, R.; Abakumov, S.; Vandaele, J.; Kudo, T.; Masuhara, H.; Scheblykin, I. G.; Hofkens, J.; Rocha, S. Fast-Tracking of Single Emitters in Large Volumes with Nanometer Precision. *Opt. Express* **2020**, *28* (19), 28656–28671. https://doi.org/10.1364/OE.401557.

(2) Otsu, N. A Threshold Selection Method from Gray-Level Histograms. *IEEE Trans. Syst. Man Cybern.* **1979**, *9* (1), 62–66. https://doi.org/10.1109/TSMC.1979.4310076.

(3) Thermo Fisher Scientific. *Fluorescein Equivalents in Molecular Probes Yellow-Green–Fluorescent FluoSpheres Beads—Table 6.6*. https://www.thermofisher.com/be/en/home/references/molecular-probes-the-handbook/tables/fluorescein-equivalents-in-our-yellow-green-fluorescent-fluospheres-beads.html (accessed 2026-01-23).
